# TGF-β1 Down-Regulation of NKG2D/DAP10 and 2B4/SAP Expression on Human NK Cells Contributes to HBV Persistence

**DOI:** 10.1371/journal.ppat.1002594

**Published:** 2012-03-15

**Authors:** Cheng Sun, Binqing Fu, Yufeng Gao, Xiaofeng Liao, Rui Sun, Zhigang Tian, Haiming Wei

**Affiliations:** 1 Institute of Immunology, School of Life Sciences, University of Science and Technology of China, Hefei, China; 2 Hefei National Laboratory for Physical Sciences at Microscale, University of Science and Technology of China, Hefei, China; 3 Department of Liver Diseases of the Second Affiliated Hospital of Anhui Medical University, Hefei, China; Nationwide Children's Hospital, United States of America

## Abstract

The mechanism underlying persistent hepatitis B virus (HBV) infection remains unclear. We investigated the role of innate immune responses to persistent HBV infection in 154 HBV-infected patients and 95 healthy controls. The expression of NKG2D- and 2B4-activating receptors on NK cells was significantly decreased, and moreover, the expression of DAP10 and SAP, the intracellular adaptor proteins of NKG2D and 2B4 (respectively), were lower, which then impaired NK cell-mediated cytotoxic capacity and interferon-γ production. Higher concentrations of transforming growth factor-beta 1 (TGF-β1) were found in sera from persistently infected HBV patients. TGF-β1 down-regulated the expression of NKG2D and 2B4 on NK cells in our *in vitro* study, leading to an impairment of their effector functions. Anti-TGF-β1 antibodies could restore the expression of NKG2D and 2B4 on NK cells *in vitro*. Furthermore, TGF-β1 induced cell-cycle arrest in NK cells by up-regulating the expression of p15 and p21 in NK cells from immunotolerant (IT) patients. We conclude that TGF-β1 may reduce the expression of NKG2D/DAP10 and 2B4/SAP, and those IT patients who are deficient in these double-activating signals have impaired NK cell function, which is correlated with persistent HBV infection.

## Author Summary

NK cells have been viewed as the most important effectors of the initial antiviral innate immune response. Their activation depends on the integration of signals from “co-activation” receptors, and the cytotoxic effects of NK cells on target cells are tempered by a need for combined signals from multiple activating receptors, such as NKG2D and 2B4. In this study, we showed that NKG2D and 2B4 expression levels were decreased on NK cells from patients in the IT phase of HBV infection. We further demonstrated that lower levels of intracellular adaptor proteins (DAP10 and SAP) were associated with lower surface expression of NKG2D and 2B4. As a result, the synergistically co-activated signalling pathway initiated by NKG2D and 2B4 did not operate properly in IT-phase patients. We demonstrated that high levels of soluble TGF-β1 were associated with the reduction of NKG2D and 2B4 in patients. In addition, we showed that TGF-β1 causes the cell-cycle arrest of NK cells by up-regulating the levels of p15 and p21 in NK cells from IT patients. Collectively, these findings may contribute to our understanding of the immune tolerance mechanism and aid in the development of novel therapeutic methods to clear HBV infection during the initial phase.

## Introduction

Hepatitis B virus (HBV) infects more than 350 million people worldwide, accounting for over 1 million deaths annually due to immune-mediated chronic liver damage [1]–[3]. The course of HBV infection is complicated. Three phases of chronic HBV (CHB) infection are now widely accepted: 1) an immune tolerant (IT) phase, characterised by an HBV DNA concentration >200,000 IU/mL, normal alanine aminotransferase (ALT) levels, normal liver biopsy or only minimal inflammation and perinatal infection of infants born to HBsAg/HBeAg-positive mothers; 2) an immune active (IA) phase, which is also referred to as the “chronic hepatitis B phase” or the “immune clearance phase”, characterised by an HBV DNA concentration >20,000 IU/mL, elevated ALT levels and active hepatic inflammation on biopsy; and 3) an inactive (IN) phase, characterised by HBV DNA levels <2000 IU/mL, normal ALT levels and minimal or absent hepatic inflammation [4]. During the IA phase, the immune system of the host recognises the virus as foreign and initiates the immune clearance response, which results in hepatocyte damage. After one or more episodes of reactivation to the IA phase, patients begin the IN phase [5]–[7]. Patients in the IT phase have normal ALT levels and elevated levels of HBV DNA, commonly well above 1 million IU/mL; this phase can last anywhere from a few to >30 years [8]. The IT phase has been suggested to occur most frequently in patients who were infected via perinatal transmission from HBeAg-positive mothers [9]. In China, approximately 2 million infants are infected with HBV via perinatal transmission from HBsAg/HBeAg-positive mothers annually, and many of them are at high risk of developing chronic liver inflammation resulting in cirrhosis and hepatocellular carcinoma (HCC) in later life [10]–[13]. The risk of developing HCC with HBV infection is higher in East Asian countries than in Western countries, possibly due to the frequency of earlier viral infection [14]. During the IT phase of CHB infection, the virus evolves strategies to evade immune clearance in the majority of patients. However, the tolerance mechanism of the IT phase has not been widely studied. Due to their normal ALT levels, there is no available treatment to reduce the very high HBV DNA levels or alleviate psychological pressure in IT-phase patients. The development of an anti-HBV therapy for such patients will require insight into the mechanisms of HBV persistence.

The innate immune system provides the first line of defence in antiviral responses and activates adaptive immune responses. NK cells have been viewed as the most important effectors of the initial antiviral innate immune system [15]–[16]. Previous investigations have demonstrated that NK cells may be particularly important in patients with CHB. NK cells are highly enriched in the liver, and the site of HBV replication, and they are partially functionally tolerant in CHB [13], [17]. The substantial quantity of NK cells in the liver suggests that they act as “watcher cells”, surveying the liver for indications of cellular stress and implying that HBV has to evade NK cell-mediated immune responses to establish a persistent viral infection. The evidence shows that the cytotoxic capacity of NK cells is retained. Moreover, the activation of NK cells and the secretion of IFN-γ are strongly inhibited during CHB infection [18]. Blockage of IL-10 with or without TGF-β1 can restore the capacity of NK cells to produce the antiviral cytokine IFN-γ in CHB patients [19]. NKG2D, the activating receptor of NK cells, is constitutively expressed on human NK cells and CD8^+^ T cells [20]. The importance of the NKG2D pathway is highlighted by evidence that tumours and viruses have developed distinct escape mechanisms to avoid NKG2D-mediated recognition [21]–[26]. The signalling lymphocyte activation molecule (SLAM)-related receptor 2B4 is predominantly expressed on human NK cells and CD8^+^ T cells. The immunoregulatory role of 2B4 as an activating or inhibitory receptor depends on three factors: 1) surface expression, because costimulatory qualities are associated with low expression and inhibitory qualities are associated with high expression; 2) the coexpression of additional inhibitory molecules; and 3) the presence of the intracellular adaptor protein SLAM-associated protein (SAP) [18], [27]–[30]. NKG2D and 2B4 are the main triggering receptors of NK cells [31]. Many studies have provided evidence for a functional dichotomy in patients with chronic HBV that may contribute to virus persistence [32]. NK cell-mediated cytotoxicity is efficiently initiated by the NKG2D activation signal on NK cells. NKG2D recognises pressure-induced antigen signals on a target cell, whereas 2B4 receives the costimulatory signal. Therefore, these two molecules play a key role in NK cell activation and function. The tolerance mechanism of HBV persistence and the contribution of the NKG2D/DAP10 and 2B4/SAP pathways to the control of persistent HBV infection are unclear. Here, we show that NKG2D/DAP10 and 2B4/SAP are down-regulated on circulating NK cells and are associated with the impaired functionally of NK cells in IT-phase patients. Moreover, this defect is mediated by TGF-β1, which causes NK cell-cycle arrest by inducing high expression of p15 and p21. These findings may contribute to our understanding of immune tolerance mechanisms and may aid in the development of novel therapeutic methods to clear the viral infection during the initial phase.

## Results

### Expression levels of NKG2D and 2B4 on circulating NK cells were decreased during the IT phase of hepatitis B virus infection

**Figure 1:**
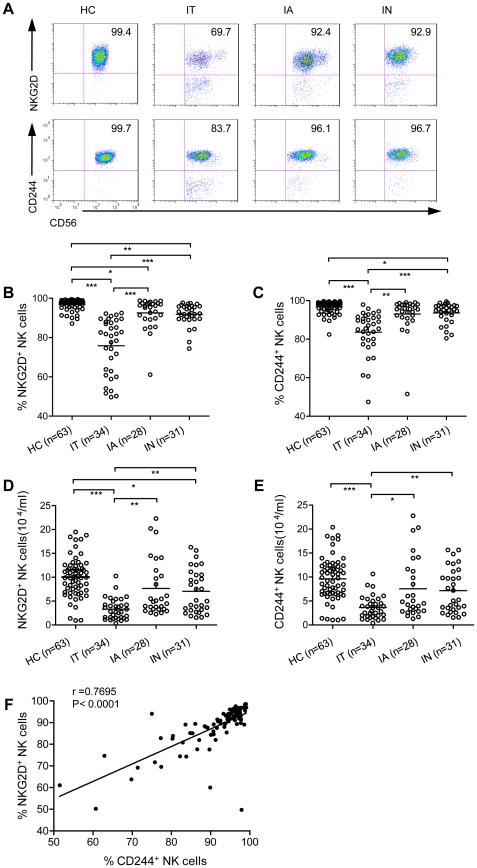
NKG2D and 2B4 expression is decreased on circulating NK cells during the IT phase of HBV infection. Immunofluorescent staining of human cells was performed to investigate the expression of NKG2D and 2B4 on NK cells, as gated by FACS analysis. Horizontal bars denote the means. (A) NKG2D and 2B4 expression on total CD3^+^CD56^−^ NK cells within the lymphocyte gate from a representative healthy control or CHB patient. (B and C) Differential NKG2D and 2B4 expression on total NK cells within the lymphocyte gate in HC and samples from patients in the IT, IA and IN phases. (D and E) Absolute counts of NKG2D^+^ and 2B4^+^NK cells in HC, IT, IA and IN. The expression of NKG2D and 2B4 was lower, both by percentage and absolute count, on IT NK cells than on NK cells from both healthy controls and other chronic patients. (F) Inverse correlation between serum 2B4 levels and NKG2D levels on NK cells in HBV patients. Pearson's correlation coefficient: r = 0.7695, P<0.0001. * p<0.05 and *** p<0.001.

The multiple functions of NK cells, such as cytotoxicity and cytokine secretion, can be induced through interactions between inhibitory and activating NK receptors and their respective ligands [15]. NKG2D is constitutively expressed on NK cells and is one of the main triggering receptors of NK cells. NK cell-mediated cytotoxicity is efficiently initiated by engaging the NKG2D-DAP10 receptor complex on NK cells [31]. To explore the effector potential of NK cells during persistent HBV infection, we first analysed the frequency of NKG2D expression on NK cells in 93 patients with HBV infection compared to 63 healthy gender- and age-matched controls. Due to the restricted availability of fresh, persistently HBV-infected liver samples from which to isolate infiltrating NK cells, we examined the expression of NKG2D on circulating NK cells. HBV-infected patients were classified into three phases based on their natural histories: the IT phase, the IA phase and the IN hepatitis B phase [4], [33]–[34]. The proportion of circulating NKG2D^+^ NK cells was significantly decreased in patients in the IT phase relative to healthy controls (P<0.0001) and patients in either the IA phase (P = 0.04) or the IN (P = 0.001) phase (Figure 1.A, B). The proportion of total NK cells in IT-phase patients was also lower than in CHB patients in other phases or in healthy controls (Figure S3.B). More importantly, we observed that both the percentage of circulating NKG2D-expressing NK cells and the absolute count of NKG2D^+^ NK cells were significantly lower in IT-phase patients than in healthy controls (P<0.0001) or patients in the IN (P = 0.019) but not patients in the IA (P = 0.303) (Figure 1.D).

To explore whether other activating NK receptors were expressed at low levels in the IT patients, we quantified another NK cell-activating receptor, 2B4, using flow cytometry. The proportion of circulating 2B4^+^ NK cells displayed significantly lower levels in the IT patients than in healthy controls (P<0.0001) and in patients in the IN (P = 0.022) (Figure 1.A, C). Similarly, the absolute number of 2B4-expressing NK cells in patients in the IT phase was lower than in healthy controls (P<0.0001) (Figure 1.E). Upon further analysis, a linear relationship was observed between the percentage of 2B4 and NKG2D on NK cells (r = 0.7695, P<0.0001) (Figure 1.F). We also analysed the frequency of the expression of other NK cell activation receptors (NKp30, NKp44, NKp46, CD16, CD27 and CD226) on circulating NK cells from healthy controls and CHB patients, but there were no significant differences in their expression levels on NK cells between patients and healthy controls (Figure S2.A,B). The levels of NKG2D and 2B4 did not correlate with age, HBV viral load or ALT/AST levels (data not shown), and NKG2D (P = 0.0112) and 2B4 (P = 0.0101) expression levels were lower in females than males (Figure S3.A).

### NKG2D and 2B4 reductions in IT patients are associated with deficient NK cell function

**Figure 2:**
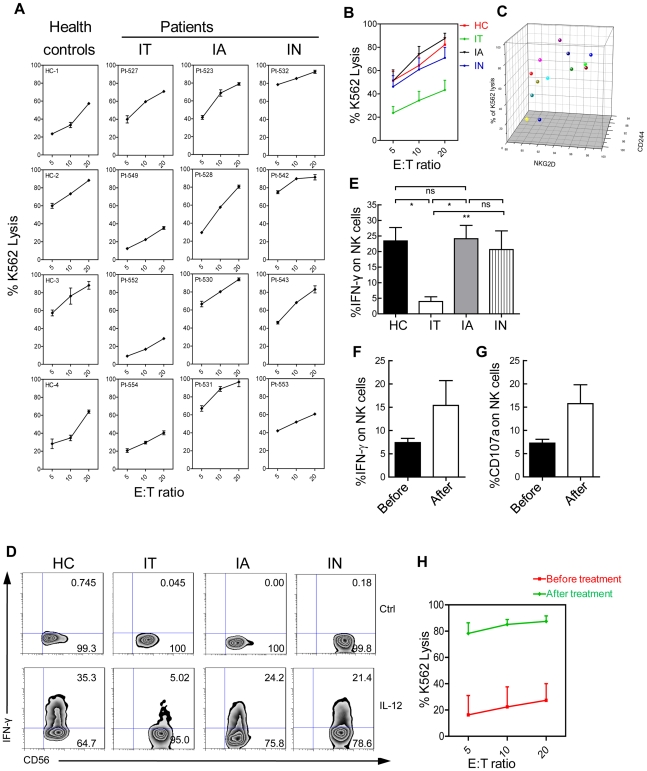
Deficient NK cell function in IT patients. (A) Deficient NK cell cytotoxicity from IT patients. NK cell cytotoxicity was assessed in primary NK cells from IT, IA, and IN patients and healthy controls using a ^51^Cr-release assay with K562 target cells. (B) Results show the mean ± SEM values in 19 patients and 5 controls. Values for IT patients were less than controls (P = 0.0384). (C) Positive correlation between NK cell cytotoxicity and the percentage of NK cells expressing NKG2D and 2B4. (D) Fresh PBMCs were stimulated with/without IL-12 as described in the Materials and Methods. After 16 h, IFN-γ production was determined using flow cytometry by gating on CD3^−^CD56^+^ NK cells. A representative dot plot displaying intracellular IFNγ staining in NK cells with the subsets indicated is shown. (E) Cumulative data are shown. (F, G) Fresh PBMCs from patients before treatment (black) or after treatment (white) were stimulated with IL-12 for 16 h. IFNγ (F) and CD107a (G) production was determined using flow cytometry by gating on CD3^−^CD56^+^ NK cells. (H) Induction of *ex vivo* NK cell cytotoxic activity after *in vivo* administration of antiviral treatment with nucleoside analogues until ALT reached normal levels. NK cytotoxicity was determined *ex vivo* by measuring the lysis of ^51^Cr-labelled target cells before treatment (red) or after treatment (green). Cumulative data are shown.

The activation of NK cells depends on the integration of signals from co-activation receptors, and the cytotoxic effects of NK cells on target cells are tempered by a request for combined signals from multiple activating receptors, such as NKG2D and 2B4 [16], [35]–[36]. Therefore, we hypothesised that these phenotypic changes might be paralleled by functional alterations in NK cells. To determine whether NK cells in IT-phase patients have an intrinsic defect in cytolytic activity, NK cell cytotoxicity was evaluated by measuring the lysis of ^51^Cr-labelled K562 cells. To ensure that there was no other cellular factor that could influence NK cell cytotoxicity, we purified NK cells from PBMCs by negative selection. IT-phase patients were deficient in K562 killing compared to healthy controls and patients in the IA or IN phase (Figure 2.A). Across multiple evaluations, the IT patients had a mean of 23.6±5.5% K562 lysis at a 5∶1 E∶T ratio compared with 51.4±7.7% for the controls (P = 0.0384). No significant difference in NK cell cytotoxicity was detectable between patients in the IA or IN phases and healthy controls (Figure 2.B). These data demonstrate that patients in the IT phase have a specific defect in NK cell cytotoxic activity. Furthermore, a positive correlation was observed between the cytotoxicity of NK cells and the percentage of NKG2D^+^NK cells (r = 0.7264) and the percentage of 2B4^+^NK cells (r = 0.4183) (Figure 2.C). To further evaluate the potential function of NK cells in IT-phase patients, we examined their capacity to produce cytokines by measuring IFN-γ expression following stimulation with IL-12. There was a significant reduction in the production of IFN-γ produced by NK cells from patients in the IT phase compared with healthy controls and IA- and IN-phase patients (Figure 2.D, E). There was also no change in T-bet expression in NK cells from patients and healthy controls (Figure S4.A, B).

To further determine whether the defect in NK cell function could be reversed during antiviral therapy, PBMCs from patients at the onset of inflammation and after treatment were stimulated with IL-12 for 16 h, and IFN-γ, and CD107a production was determined. As shown in Figure 2, the production of IFN-γ (F) and CD107a (G) was higher in NK cells from patients after treatment compared with before treatment, suggesting that NK cell function could be reversed by antiviral therapy. To further validate this hypothesis, *ex vivo* NK cell cytotoxicity against K562 cells was measured (Figure 2.H). Our results demonstrated that NK cell cytotoxic activity was strengthened after antiviral therapy. These results suggest that *in vivo* administration of nucleoside analogues partially overcomes the deficit in NK cell function in CHB patients.

### Reduced expression of DAP10 and SAP on NK cells from IT-phase patients

**Figure 3:**
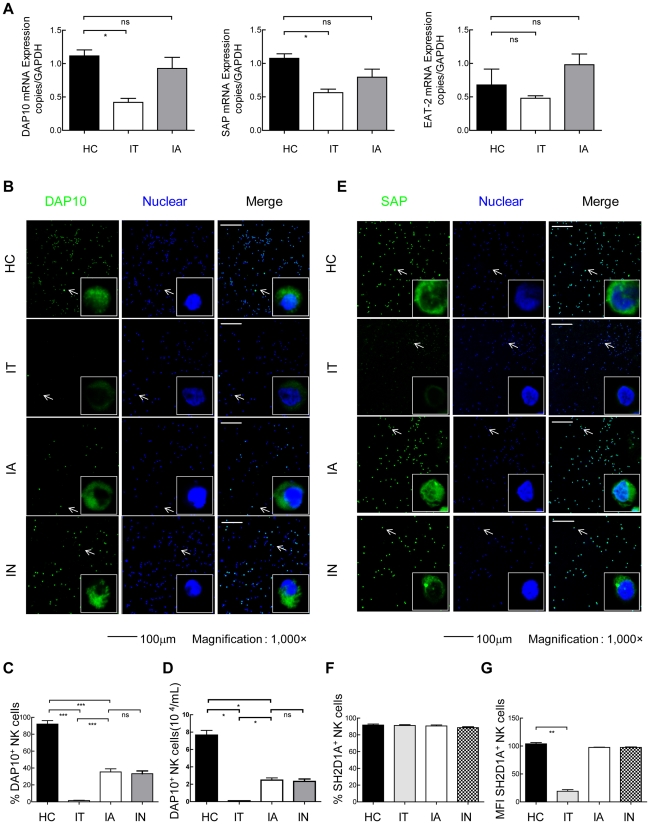
Reduced expression of DAP10 and SAP in NK cells from IT patients. (A) The mRNA expression levels of DAP10, SAP and EAT-2 in NK cells were investigated in cells from HBV patients and healthy controls. Confocal immunofluorescence images of DAP10 (green) (B) or SAP (green) (E) staining in primary NK cells in HC, IT, IA and IN patients, showing the down-regulation of DAP10 and SAP in NK cells from IT patients compared with healthy controls and other chronic patients in cells also co-stained with DAPI. (C and F) Graph showing the percentages of NK cells expressing DAP10 or SAP. (D) Absolute counts of DAP10^+^ NK cells in HC, IT, IA and IN. (G) Differential MFI of SAP^+^ NK cells from HC and IT-, IA- and IN-phase patients. The results are representative of 1000 cells. Scale bars in the immunofluorescence images represent 100 µm. The data are represented as the means ± SEM. *** P<0.001. Original magnification: 20× (B, E).

**Figure 4:**
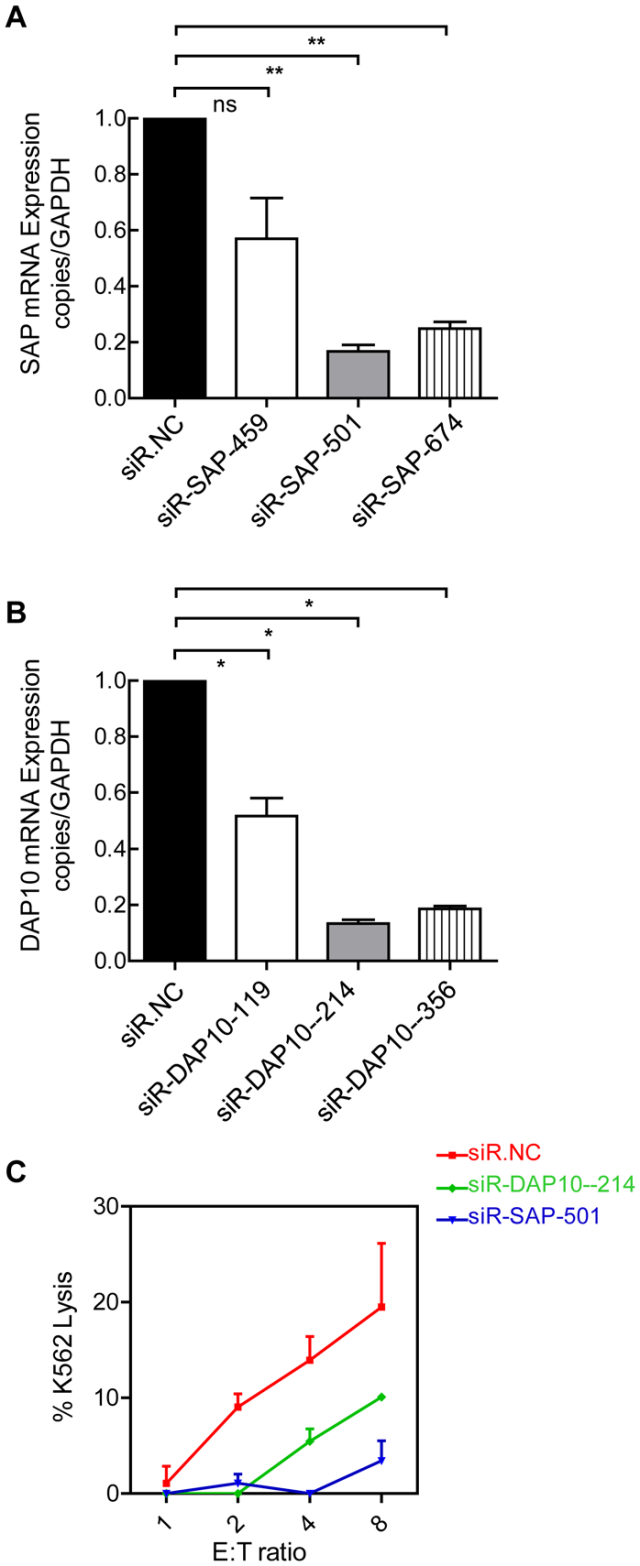
Deficient NK92 cell function from siR-DAP and siR-SAP. (A, B) Real-time PCR analysis of the mRNA expression levels of DAP10, SAP on NK92 cells were investigated 48 h after transfection with either an siRNA negative control (siR.NC), DAP10 siRNA (siR-DAP) or SAP siRNA (siR-SAP). (C) NK92 cell cytotoxicity was assessed 20 h after transfection with siR.NC, siR-DAP or siR-SAP using a ^51^Cr-release assay with K562 target cells. The data are shown as the mean ± SE, siR-DAP had a mean of 10.1%±0.3% and siR-SAP had a mean of 3.4%±1.2% K562 lysis at a 8∶1 E∶ T ratio compared with 19.5%±3.7% for the controls. Values for siR-DAP and siR-SAP were lower than for the negative control (P = 0.027 and P = 0.002, respectively).

We observed that both the percentage and the absolute count of NKG2D and 2B4 were decreased on circulating NK cells during IT-phase HBV infection. We then asked whether the decreased expression of activating receptors on NK cells was paralleled by functional alterations. There is a consensus that the NKG2D-DAP10 receptor complex on human NK cells efficiently initiates cell-mediated cytotoxicity [31]. When NK cells encounter target cells expressing CD48, the 2B4-SAP–Fyn complex is responsible for the activation of NK cells. To test this hypothesis, we measured the expression of the intracellular adaptor protein DAP10 and the two members of the SAP family of adaptors that are expressed in humans: SAP (also known as SH2D1A or DSHP) and Ewing's sarcoma-activated transcript-2 (EAT-2, also known as SH2D1B). The mRNA expression levels of DAP10, SAP and EAT-2 in NK cells were investigated in HBV patients and compared to healthy controls. As shown in Figure 3.A., our results demonstrated that the mRNA expression levels of DAP10 and SAP were lower in NK cells from IT patients than in those from IA patients and healthy controls; there were no differences between IA patients and healthy controls. Moreover, there were no significant differences in EAT-2 mRNA expression levels in NK cells between patients and healthy controls. To further investigate the signalling potential of activating NKRs within NK cells, we assessed the protein expression levels of DAP10 and SAP in NK cells. Due to having access to limited numbers of NK cells from blood samples, we investigated the expression of DAP10 and SAP in NK cells by immunofluorescence. As shown in Figure 3.B, we found fewer DAP10^+^ NK cells in samples from IT-phase patients than in those from patients in other phases and healthy controls. The proportion and absolute numbers of circulating DAP10^+^ NK cells were also lower in the IT patients compared with the patients in other phases and healthy controls (Figure 3.C, D). No significant differences in DAP10^+^ NK cells were detectable between patients in the IA and IN phases (Figure 3.C, D). Upon further analysis, the percentages of SAP-expressing NK cells were similar in HBV patients and healthy controls (>95% of NK cells in all four groups). However, a decreased MFI was detected in SAP^+^ NK cells from IT patients compared with healthy controls and IA or IN patients (Figure 3.G). No significant difference in SAP^+^ NK cells was detectable between patients in the IA and IN phases (Figure 3.F, G). Furthermore, to verify whether SAP proteins were reduced or not, we examined the expression of SAP in NK cells using western blotting (Figure S5). Our new data demonstrated that considerably low levels of SAP on NK cells from IT patient samples and relatively low levels of SAP in IA, IN patients compared with healthy controls. These data suggest that the loss of NKG2D^+^ and 2B4^+^ NK cells in IT patients may be accompanied by the loss of DAP10^+^ and SAP^+^ NK cells. To verify whether this phenomenon was associated with functional consequences, we specifically silenced DAP10 (si-DAP10) or SAP (si-SAP) in NK92 cells using RNA interference (RNAi). As shown in Figure 4A, B, the mRNA expression levels of DAP10 and SAP on NK92 cells were significantly lower after transfection with either siRNA. Furthermore, we chose siR-SAP-214 and siR-DAP-501 to investigate the functional consequences. As expected, compared with NK92 cells transfected with the negative control siRNA (siR.NC), NK92 cells transfected with either DAP10 siRNA (siR-DAP) or SAP siRNA (siR-SAP) resulted in a much stronger deficiency in NK cell cytotoxicity (Figure 4.D). These results demonstrate that DAP10 and SAP, at least in part, mediate the effects of NK92 cell cytotoxicity.

### Down-regulation of the synergistic activation of Ca^2+^ flux by co-crosslinking NKG2D and 2B4 in primary NK cells from IT patients

**Figure 5:**
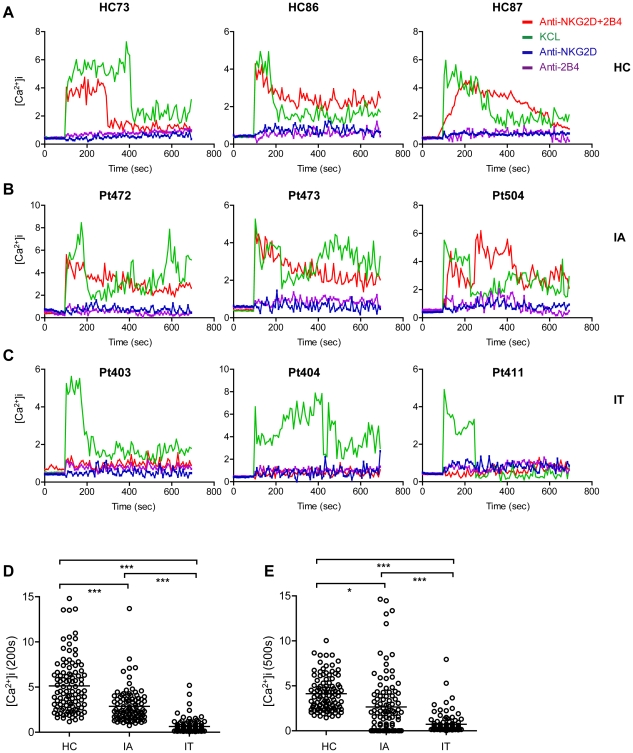
Ca^2+^ flux down-regulation is induced by synergy between NKG2D and 2B4 in NK cells from IT patients. Freshly isolated, resting NK cells from the peripheral blood of HC (A), IA (B) and IT (C) patients were loaded with Fluo-4 and Fura Red, and preincubated with mAbs specific for NKG2D (blue), 2B4 (purple) or both (red) on ice for 30 min. Cells were washed, resuspended in cold HBSS with 1% FBS, and prewarmed at 37°C. Fluorescence was measured by Zeiss 510 confocal microscopy. Sixty seconds after the beginning of each scan, secondary F(ab')^2^ goat anti–mouse IgG or KCL (green) was added to each chamber. Traces of the Fluo-4/Fura Red ratios of the representative NK cells are shown. Fluo-4/Fura Red ratios are plotted as a function of time. Green lines show activation with the isotype control (KCL). Blue and purple lines show activation by the single receptors. Red lines show activation by the combination of both receptors. The experiment shown is representative of five independent experiments. The Ca^2+^ mobilisation induced by NKG2D and 2B4 synergy was measured in >100 NK cells from representative healthy controls or patients at 200 (D) and 500 s (E).

To evaluate the functional consequences of the reduction of the double-activating signals (NKG2D and 2B4), secondary cross-linking goat anti-mouse F(ab')^2^ antibody was added to NK cells obtained from fresh peripheral blood samples and preincubated with activated mAbs for NKG2D and 2B4 to investigate the mobilisation of Ca^2+^ triggered by NKG2D and 2B4 synergy [37]. Because the supply of freshly isolated NK cells from patients and healthy controls is quite limited and because *in vitro* expansion of NK cells in the presence of cytokines changes their signalling properties, we performed confocal microscopy experiments to follow intracellular Ca^2+^ responses in 500 NK cells after the receptors were co-crosslinked. Recordings of NK cells revealed that NKG2D and 2B4 cross-linking elicited distinct responses in cells from different sources (Figure 5). In the NK cells from healthy controls and IA-phase patients, cross-linking induced a sharp and sustained increase in intracellular Ca^2+^ (Figure 5.A, B). Notably, in NK cells from IT-phase patients, there was no significant increase in intracellular Ca^2+^ flow (Figure 5.C). The Ca^2+^ mobilisation induced by NKG2D and 2B4 synergy in >100 NK cells from representative healthy controls or patients at 200 (Figure 5.D) and 500 s (Figure 5.E) was analysed. Altogether, these results suggest that the synergistic co-activation signalling pathway activated by NKG2D and 2B4 may not operate properly in IT-phase patients due to the reduction in NKG2D and 2B4 expression in NK cells.

### Soluble TGF-β1 was associated with the reduction in NKG2D and 2B4

**Figure 6:**
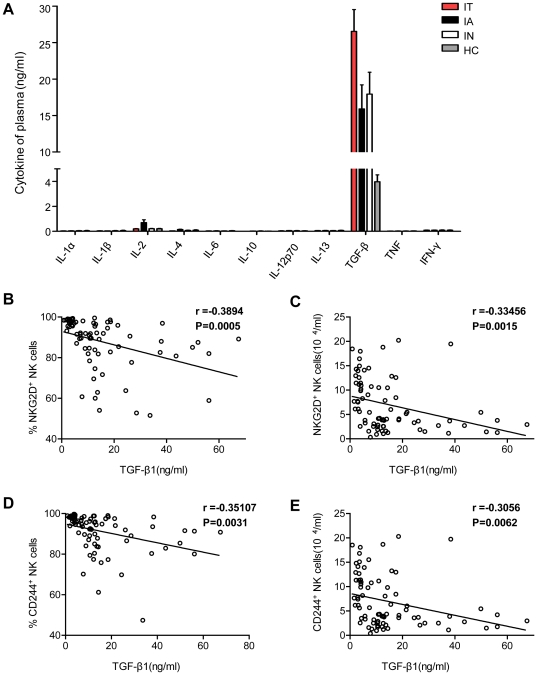
Soluble TGF-β1 is associated with reductions in NKG2D and 2B4. (A) Circulating concentrations of multiple cytokines detected in serum samples taken from healthy controls and HBV patients assayed by CBA (IL-1α, IL-1β, IL-2, IL-4, IL-6, IL-10, IL-12, p70, IL-13, TNF and IFN-γ) and sandwich ELISA (TGF-β1). Significance testing was performed using the Mann-Whitney U test. (B, D) Inverse correlations between serum TGF-β1 levels and the percentage of NK cells expressing NKG2D and 2B4. (C, E) Inverse correlation between serum TGF-β1 levels and the absolute counts of NKG2D- and 2B4-expressing NK cells. Pearson's correlation coefficients are shown.

Surface expression of NKG2D has been shown to be down-regulated by TGF-β and IL-21 [38]–[40]. During chronic HCV infection, TGF-β down-modulates the expression of NKG2D on NK cells, leading to the impairment of their function [26]. Therefore, to verify whether there are also elevated levels of TGF-β1 in patients with persistent HBV infection, we used a cytometric bead array (CBA) inflammation kit and ELISA technology to simultaneously quantify the presence of multiple cytokines in sera from HBV patients and healthy controls. The cytokines analysed were IL-1α, IL-1β, IL-2, IL-4, IL-6, IL-10, IL-12p70, IL-13, TNF, IFN-γ and TGF-β1. The peak concentrations of TGF-β1 in IT patients were far in excess of those observed for the other ten cytokines; this cytokine was also present at significantly higher concentrations than those measured in healthy controls and patients in other phases of infection (Figure 6.A). In addition, an inverse linear relationship was observed between TGF-β1 concentration and the percentage of circulating NKG2D-expressing NK cells (Figure 6.B, C). An identical relationship was identified with the proportion of 2B4-expressing NK cells (Figure 6.D, E). These data demonstrate a correlation between the down-regulation of activating NK receptors and high levels of TGF-β1 and suggest a role for the influence of soluble TGF-β1 on NK cells in IT-phase HBV patients.

### Anti-TGFβ1 partially restored surface expression of NKG2D and 2B4 on NK cells

**Figure 7:**
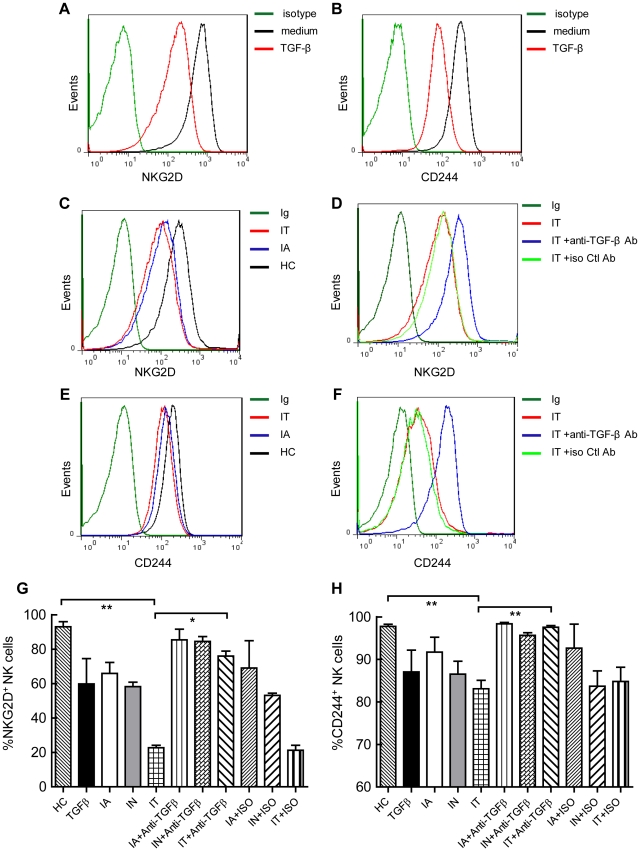
Anti-TGF-β1 partially restores the surface expression of NKG2D and 2B4 on NK cells. NK cells from healthy control peripheral blood were preincubated with TGF-β1. NKG2D (A) and 2B4 (B) expression on NK cells was monitored at 72 h by flow cytometry. Histograms correspond to NK cells from one representative donor treated with the isotype controls (green lines), medium alone (RPMI 1640 supplemented with 10% FBS in the presence of IL-15 (10 ng/ml) and IL-2 (100 U/ml)) (black lines) and TGF-β1 (1 ng/ml) (red lines). NK cells from healthy controls were cultured with healthy control sera (black lines), sera from IA-phase patients (blue lines) or sera from IT-phase patients (red lines) for 3 days. Surface expression of NKG2D (C) and 2B4 (E) were assessed at 72 h using flow cytometry. Histograms showing NKG2D (C) and 2B4 (E) surface expression on NK cells from one representative donor out of three studied are shown. Freshly isolated NK cells from healthy controls co-cultured with sera from IT-phase patients alone (red lines) or with anti-TGF-β1 Ab (blue lines) or isotype control Ab (green lines). The expression levels of NKG2D (D) and 2B4 (F) were analysed using FACS. The results are representative of three independent experiments. Cumulative data are shown (G, H).

Down-modulation of expression is associated with elevated levels of TGF-β1 and has also been observed in cancer patients [39], [41]–[45]. Therefore, we hypothesised that TGF-β1 might modulate NKG2D surface expression. We investigated whether the altered expression of activating NK cell receptors was induced by TGF-β1. To test this possibility, we used NK cells from healthy control peripheral blood preincubated with TGF-β1 for 3 days. The percentage of NKG2D and 2B4 levels on NK cells, as monitored by flow cytometry, were markedly down-regulated (Figure 7A, B). Fresh NK cells were also cultured with sera from healthy controls and patients in different immune states. After 3 days, the surface expression of NKG2D on NK cells preincubated with sera from IT patients (Figure 7.C, red lines) was markedly lower than on NK cells preincubated with sera from healthy controls (black lines). Furthermore, anti-TGF-β1 partially restored the expression of NKG2D on NK cells co-cultured with sera from IT-phase patients (Figure 7.D, blue lines). In contrast, there were no significant changes in NK cells preincubated with isotype control Abs (green lines) (Figure 7.D). In the same cell culture experiments, NK cells isolated from healthy controls were stimulated with TGF-β1 or sera from various sources. The proportion of 2B4 levels on NK cells decreased when co-cultured with TGF-β1 or sera from IT patients (Figure 7.B, E, red lines). Similarly, the expression levels of 2B4 recovered when the incubation was performed in the presence of a neutralising anti-TGF-β1 antibody (Figure 7.F, blue lines). Cumulative data shown in Figure 7.G, H indicate that the percentage of NKG2D and 2B4 levels on NK cells decreased significantly after co-culture with sera from IT patients, and minor reductions were observed when NK cells were cultured with sera from IA- or IN-phase patients. The expression of NKG2D was partially restored and 2B4 expression recovered when NK cells were incubated with neutralising anti-TGF-β1 antibody. There were no significant changes in NK cells preincubated with isotype control Abs. Taken together, these data indirectly suggest that the expression levels of NKG2D and 2B4 on NK cells were suppressed by high levels of TGF-β1 in IT-phase patients.

### Anti-TGFβ1 partially restored Ca^2+^ flux in NK cells from healthy controls incubated with sera from IT patients

**Figure 8:**
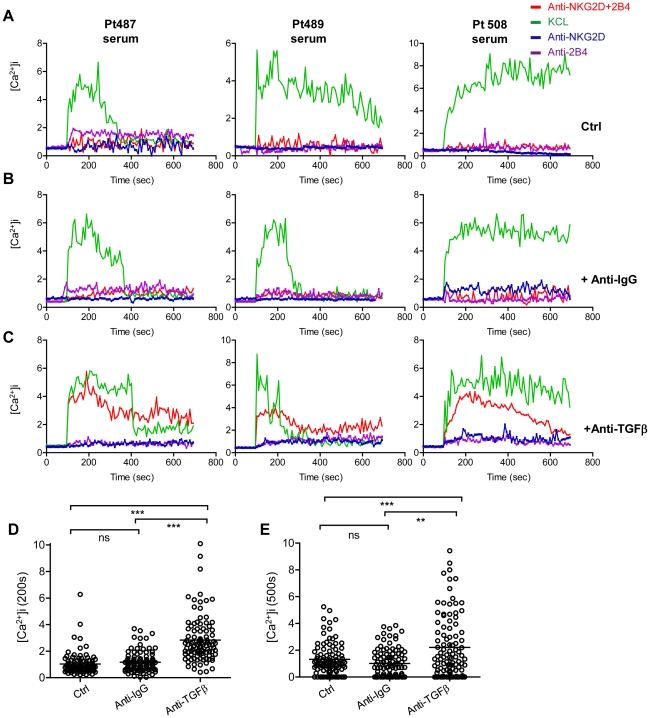
Anti-TGF-β1 partially restores Ca^2+^ flux in NK cells from healthy controls incubated with IT patient serum. Freshly isolated resting NK cells from healthy control peripheral blood were preincubated with sera from IT-phase patients (A), isotype control Abs (B) or anti-TGF-β1 Abs (C) and stimulated with NKG2D and/or 2B4, as shown in Figure 3. Ca^2+^ flux was analysed using a Zeiss 510 confocal microscope. The Ca^2+^ mobilisation induced by NKG2D and 2B4 synergy was measured in >100 NK cells from representative healthy controls or patients at 200 (D) and 500 s (E). The experiment shown is representative of 5 independent experiments.

During chronic HCV infection, TGF-β1 down-modulates the expression of NKG2D on NK cells, leading to the impairment of their function [26]. Inhibition of TGF-β1 restores the ability of NK cells from both the peripheries and livers of patients with CHB infection to produce antiviral IFN-γ [19]. Anti-TGF-β1 partially restored NKG2D and 2B4 surface expression on NK cells from IT patients. We then postulated that high levels of TGF-β1 might affect the functions of NK cells in IT patients. To investigate this hypothesis, we used *in vitro* experimental models. First, NK cells obtained from healthy controls were stimulated with sera from IT patients plus anti-TGF-β1 antibodies or isotype control Abs for 72 h to imitate a physiological state. The Ca^2+^ mobilisation induced by synergism between NKG2D and 2B4 on NK cells was then assessed. As observed in Figure 8.A, sera from IT-phase patients abrogated the Ca^2+^ mobilisation potential of NK cells from healthy controls. Additionally, anti-TGF-β1 partially restored Ca^2+^ flux in NK cells from healthy controls that had been incubated with sera from IT patients (Figure 8.C). Recordings of NK cells incubated with sera from different patients and anti-TGF-β1 Abs revealed distinct responses. In some patients (Pt508), NKG2D+2B4 cross-linking induced a sharp, oscillating rise in intracellular Ca^2+^. In other patients (Pt487, 489, and others) lower, transient peaks were observed (Figure 8.C). The Ca^2+^ mobilisation induced by NKG2D and 2B4 synergy in >100 NK cells from representative groups at 200 (Figure 8.D) and 500 s (Figure 8.E) was analysed. Overall, a rise in Ca^2+^ flux induced by NKG2D and 2B4 synergism was observed when anti-TGF-β1 Ab was added.

### TGF-β1 inhibited the cell-cycle in NK cells *in vitro*

**Figure 9:**
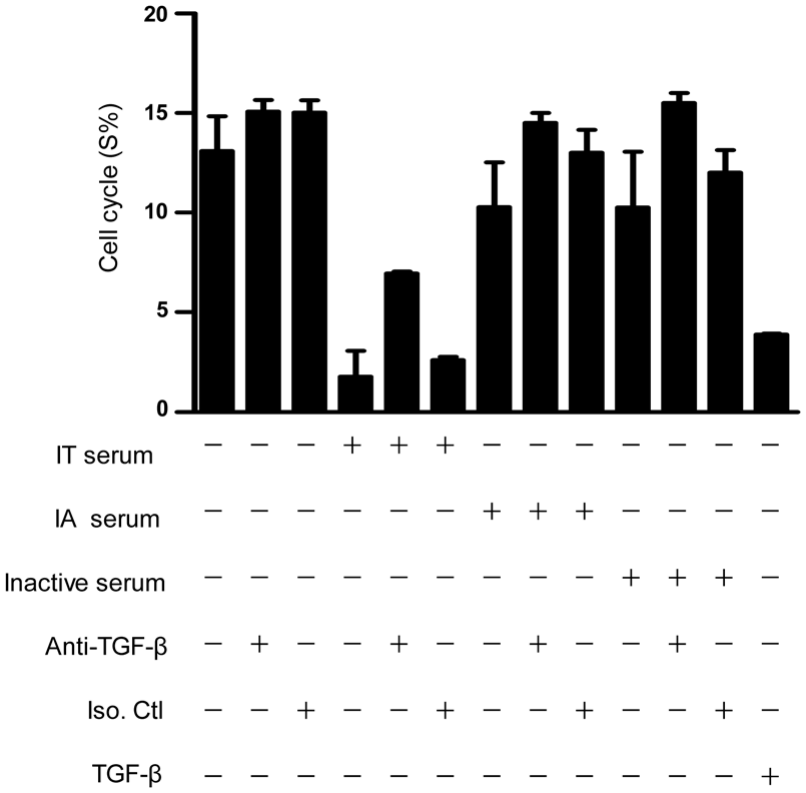
TGF-β1 inhibits the NK cell cycle *in vitro*. NK cells from healthy controls were preincubated with IT serum, IA serum, IN serum or anti-TGF-β1Ab. The proportion of cells in the G1 and S phases of the cell cycle was determined using flow cytometry, which demonstrated that TGF-β1 inhibited cell proliferation and induced G1-phase arrest. * P<0.05, ** P<0.01, and *** P<0.001.

Interestingly, four activating NK cell molecules (NKG2D/DAP10 and 2B4/SAP) have been found to be expressed at low levels in IT patients, and this phenotype was associated with high levels of TGF-β1, reminiscent of the cell-cycle arrest induced by TGF-β1. Therefore, we next examined whether high levels of TGF-β1 in IT patients could facilitate cell-cycle arrest in NK cells. An important role of TGF-β1 involves restricting the growth of neuronal, epithelial and hematopoietic cells. Key elements of this mechanism are the expression of the cyclin-dependent kinase inhibitors p15^INK4b^ and p21 (also called WAF1/CIP1) [46]–[47]. These proteins play an important role in restraining cell-cycle progression. Substantial evidence indicates that TGF-β1 participates in blocking the development of T-lineage acute lymphoblastic leukaemia by suppressing T-cell proliferation [48]. It has also been shown that the effect of TGF-β1-mediated depression of NKG2D surface expression may only depend on the transcription levels of NKG2D [39]. Due to the limited supply of fresh NK cells from patients, NK cells from healthy controls were stimulated with TGF-β1 or serum from various sources (IA, IT and IN patients) in the presence or absence of anti-TGF-β1Ab. Strikingly, NK cells preincubated with serum from IT patients exhibited both significantly decelerated cell proliferation and G1 cell-cycle arrest (Figure 9). Conversely, preincubation of NK cells with sera from IT patients plus anti-TGF-β1Ab resulted in the restoration of cell proliferation and a decrease in the cell population in G1 phase (Figure 9).

### p21 and p15 were elevated in IT patients and arrested the NK cell cycle

**Figure 10:**
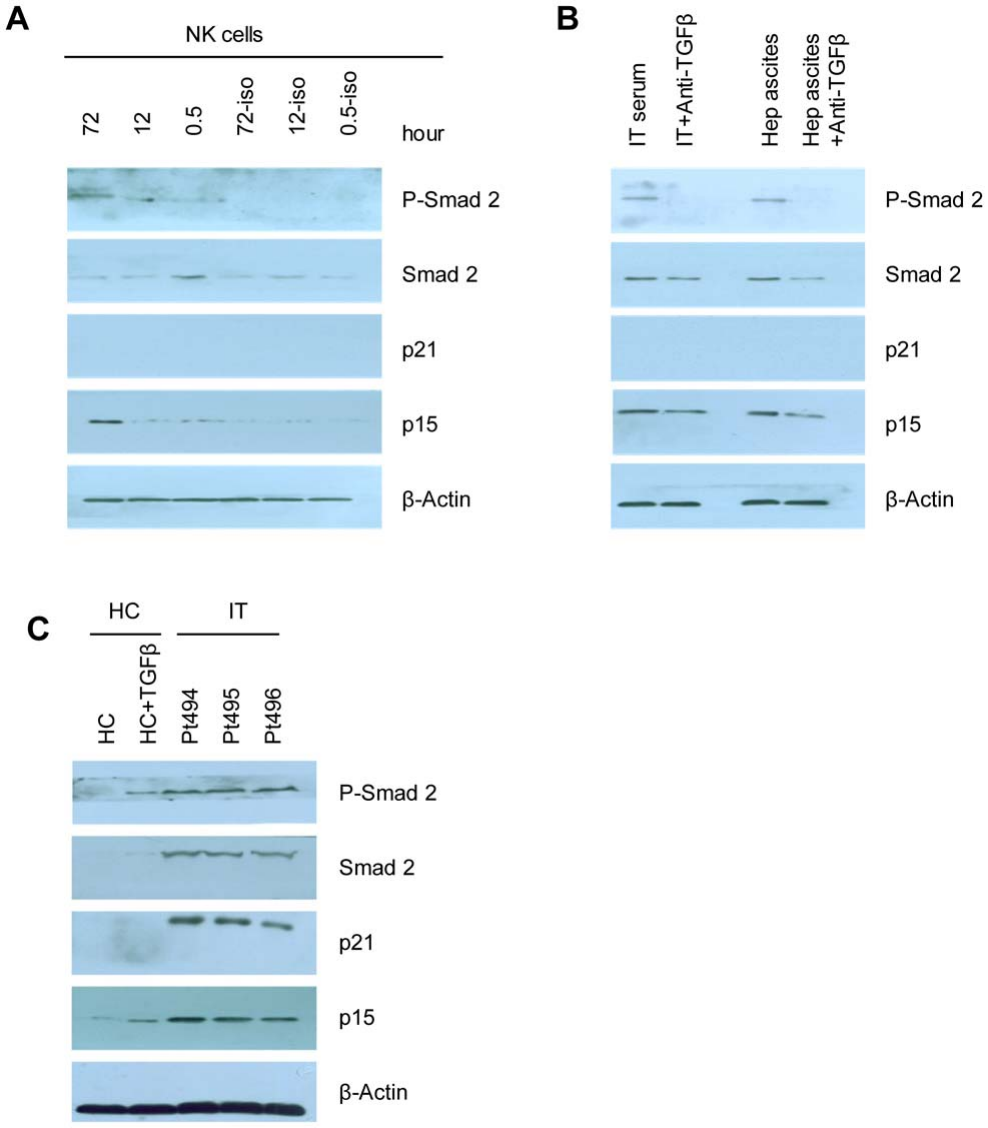
p21 and p15 are elevated in IT patients and induce the arrest of the NK cell cycle. (A) NK cells were stimulated for 30 min, 12 h and 72 h with TGF-β1. Total cell lysates were analysed using immunoblotting with antibodies against Smad-2, Smad-2P, p15 and p21. Data are representative of three independent experiments. (B) NK cells were stimulated for 72 h with IT serum or hepatitis ascites in the absence (−) or presence (+) of TGF-β1 Ab. NK cells were lysed, and the lysates were immunoblotted with Abs against Smad-2, Smad-2P, p15 or p21. Data are representative of three independent experiments. (C) Freshly isolated NK cells were obtained from the peripheral blood of healthy controls and IT-phase patients and analysed by western blotting. The results for Smad-2, Smad-2P, p15 and p21 are shown. Data are representative of at least four independent experiments.

The TGF-β-Smad signal-transduction pathway is an important tumour growth-suppressor pathway. Binding of TGF-β to the functional TGF-β1RII in NK cells induces the phosphorylation of the signalling molecule Smad2 and initiates signalling in the TGF-β-Smad pathway [46]–[47], [49]. The cyclin-dependent kinase inhibitors p15 and p21 play important roles in restraining cell cycle progression [46]. To further substantiate our finding that high levels of TGF-β1 inhibited the NK cell cycle in IT patients, we examined the expression of Smad-2, phosphorylation of Smad2 (Smad-2P) and expression of p15 and p21 in NK cells using western blotting. First, NK cells were stimulated for 30 min, 12 h and 72 h with TGF-β1. As shown in Figure 10.A, TGF-β1 was able to induce Smad-2P in NK cells from controls at 30 min and 72 h. p21 was undetectable in NK cell extracts, and p15 was able to be induced only after a 72-h incubation with TGF-β1. Next, NK cells were preincubated with sera from IT patients or with hepatitis ascites with or without anti-TGF-β1Ab for 72 h. As expected, sera and ascites were able to induce the expression of P-Smad-2 and p15 but had no effect on the expression of p21. In comparison, our analysis of NK cells preincubated with anti-TGF-β1 Ab confirmed the absence of P-Smad-2 and the reduced levels of Smad2 and p15 in the absence of TGF-β1 signalling (Figure 10.B). We then assessed the endogenous status of Smad2, P-Smad-2, p15 and p21 in patients. Fresh NK cells obtained from the peripheral blood of three IT patients were analysed using western blotting (Figure 10.C). Relatively high levels of Smad-2P, Smad2 and p15 on NK cells were observed in IT patient samples. In particular, the levels of p21 exhibited considerable enhancement in IT patients (Figure 10.C).

Altogether, these data demonstrate that TGF-β1 signalling is responsible for the defects in NK cell phenotypes and performance in IT-phase patients, and the defective NK cells found in patients facilitate persistent HBV infection.

## Discussion

There are over 350 million persistent HBV carriers worldwide, and approximately 90% of children become chronic carriers after HBV infection [1], [3]. Immune tolerance is a serious problem in CHB carriers, who are at high risk of developing cirrhosis and HCC later in life [4], [10]. HBV persistence is thought to result from inefficiencies of innate and adaptive immune responses. NK cells are a major component of innate immunity. Accumulating evidence has suggested a role for NK cells in the fight to control persistent virus infection [15], [50]–[52]. However, the tolerance mechanisms of HBV persistence have not been well explored. For the first time (to our knowledge), we demonstrated that NKG2D/DAP10 and 2B4/SAP were down-regulated on circulating NK cells. Consequently, these NK cells were functionally impaired in IT-phase patients. The loss of these molecules was mediated by TGF-β1, which resulted in cell-cycle arrest due to the induction of p15 and p21. Our results indicated that NKG2D and 2B4 expression were decreased on circulating NK cells from IT-phase patients but not CHB patients in other phases or healthy controls. Furthermore, DAP10 and SAP, the intracellular adaptor proteins of NKG2D and 2B4 in humans, were also significantly reduced in NK cells from IT patients. It has been reported that NK cell cytotoxicity towards target cells is tempered by a request for combined signals from multiple activating receptors, such as NKG2D and 2B4 [16], [35]–[36]. To evaluate the functional consequences of the observed reduction in the proportion of NKG2D and 2B4, Ca^2+^ mobilisation triggered by the double-activating signals was analysed. Our data revealed that the down-regulation of Ca^2+^ flux was induced by synergism between NKG2D and 2B4 in NK cells from IT patients but occurred at normal levels in IA patients and healthy controls. In addition, NK cell cytotoxicity and IFN-γ production were decreased in IT patients compared to healthy controls and IA patients. Anti-TGF-β1 Abs could partially restore Ca^2+^ flux in NK cells from healthy controls incubated with sera from IT patients. Moreover, anti-TGF-β1 also restored NKG2D and 2B4 surface expression on NK cells incubated with sera from IT patients. p21 and p15 were elevated in IT patients and induced the arrest of the NK cell cycle. Taken together, these results suggest that TGF-β1 reduces NKG2D/DAP10 and 2B4/SAP expression on NK cells during persistent HBV infection and suppresses innate antiviral immunity by blocking the cell cycle, which would eventually provide an additional HBV strategy to avoid NK cell-mediated recognition.

Our results suggest that NK cells may be functionally impaired in IT patients. This conclusion is supported by at least four important findings. First, the percentage of NKG2D and 2B4 levels was lower on NK cells from patients in the IT phase compared to patients in other phases and healthy controls, which indicates an activation defect in circulating NK cells. Second, lower levels of intracellular adaptor proteins were associated with lower surface expression levels of NKG2D and 2B4, which implies that the signalling pathways leading to NK-cell activation might be impeded. Specific silencing of DAP10 or SAP led to deficient NK-cell cytotoxicity. Third, patients in the IT phase had a specific defect in NK cell cytotoxic activity. Moreover, there was a significant reduction in the production of IFN-γ by NK cells from patients in the IT phase compared to healthy controls and IA- and IN-phase patients. Fourth, NKG2D and 2B4 receptor synergy down-regulated the mobilisation of Ca^2+^ in primary NK cells from IT patients. These observations indicate that NK cells are not completely functional during the IT phase, which may contribute to the persistence of HBV infections. In our study, IT-phase HBV patients were characterised by lower levels of NKG2D and 2B4 compared with healthy individuals. To our knowledge, this is the first report of reduced expression of NKG2D/DAP10 and 2B4/SAP in IT patients, thus indicating that the function of NK cells was impaired due to deficiencies in the double-activating signals. In this context, abundant data in cancer patients has shown that impaired NK function can be attributed to the down-modulation of activating receptors, such as NKG2D, which can be inhibited via TGF-β1 [42]–[44]. Notably, NKG2D-dependent NK cell functions are also modulated during chronic HCV infection [26]. These findings provide further evidence for our observation that NKG2D was down-regulated during the IT phase of HBV infection. The increased expression of 2B4 on virus-specific CD8^+^ T cells, both in the peripheral blood and in the liver, is believed to mediate inhibitory signalling in the absence of SAP during CHB infection [53], but this has not been evaluated in NK cells in the presence of HBV infection. Interestingly, the absence of 2B4 resulted in diminished LCMV-specific CD8^+^ T cell responses and prolonged viral persistence in mice persistently infected with LCMV. Additionally, long-lasting viral persistence was regulated by 2B4-deficient NK cells acting early in infection. These observations illustrate the value of NK cell self-tolerance to activated CD8^+^ T cells in early infection, similar to the IT phase of HBV infection; these results also demonstrate how NK cells can regulate a persistent infection that appears to be dependent on T cell responses [54]. We also analysed the frequency of NKp30, NKp44, NKp46, CD16, CD27 and CD226 expression in circulating NK cells from healthy controls and CHB patients. We observed that the frequency of NKp30 was slightly decreased in patients in the IT phase relative to the healthy controls. No statistical differences existed on other receptors between patients and healthy controls. It has been previously shown that TGF-β1 can down-regulate NKp30 and NKG2D *in vitro*, suggesting that slightly decrease of NKp30 in IT-phase patients may related to the high levels of TGF-β1 [39]. Evidence from previous studies suggested that SAP deficiency could lead to the inhibition of NK cytotoxicity in humans [28], [55]–[56]. It has also been shown that in the absence of SAP, lymphoma development would normally be eliminated by apoptosis in patients with X-linked lymphoproliferative disease [57]. However, the analysis of potential apoptosis caused by reducing expression of SAP indicated that the deficiency of SAP would not lead to the significant apoptosis of NK92 cells (Data not show). Here, we show that the expression of SAP is deficient in NK cells from patients in the IT phase, suggesting that 2B4 receptors may become inhibitory, rather than activating, during the IT phase of HBV infection due to the absence of SAP. 2B4 molecules with inhibitory functions are responsible for the inability of NK cells to kill virus-infected cells. This feature may further compromise NK cell function in IT patients in the absence of SAP, which implies a positive regulatory role for SAP during NK cell activation. Furthermore, TGF-β down-modulates NKG2D expression on NK cells, leading to the impairment of their cytolytic activity and ability to produce IFN-γ during HCV infection [26]. Therefore, we suggest that there may be a functional impairment in NK cells in patients in the IT phase of HBV infection.

T and B cells each possess a single antigen receptor, which regulates their development and activation. Signals initiated through antigen receptors need co-stimulatory molecules to augment the signals. In contrast, NK cells do not possess one dominant receptor but instead depend on synergistic co-activation by NK cell receptors to initiate effector functions. In our studies, we have observed that Ca^2+^ mobilisation cannot be induced by NKG2D and 2B4 synergy in NK cells from IT patients, providing an explanation for the dominance of the double-activating signals in controlling NK cell activation. Strikingly, the deficiencies of the double-activating signals may be significant with respect to NK-cell tolerance. TGF-β1 inhibits NK cell activity and cytotoxicity by down-regulating NKG2D, as originally proposed in various cancer fields [42]–[44]. It has also been observed that TGF-β can impair NK cell cytolytic activity and IFN-γ production during HCV infection [26]. However, the role of TGF-β1 in blocking NK-cell function during the IT phase of HBV infection has not yet been well defined. Our data demonstrate that TGF-β1 can down-regulate NKG2D and 2B4 surface expression and restrain the Ca^2+^ mobilisation triggered by NKG2D and 2B4 receptor synergy. Moreover, anti-TGF-β1 Abs can restore NKG2D and 2B4 expression and also partially restore Ca^2+^ flux. HCV infection can induce TGF-β1, which can regulate HCV RNA replication [58]. HCV-specific Th17 cells are suppressed by virus-induced TGF-β [59]–[60]. Furthermore, the HBV X protein significantly up-regulates the expression of TGF-β1 and TGF-βRII in the LX-2 human hepatic stellate cell line [61]. The HBV-encoded pX oncoprotein amplifies TGF-β signalling [62], and HBV X antigen promotes TGF-β1 activity [63]. These findings suggest that during the IT phase of HBV infection, there may be a similar mechanism by which the virus evades host-protective immune responses.

NK cell function can be regulated by TGF-β [49] and by CD4^+^ CD25^+^ regulatory T cells (Tregs) through a TGF-β-dependent mechanism in both humans and mice [64]–[65]. We surmised that in IT patients, Treg cells might be the source of high levels of TGF-β. In a similar mechanism, Treg cells expressing membrane-bound TGF-β directly down-regulated the expression of NKG2D/2B4 on NK cell surfaces and inhibited NK cell effector functions [64]. Previous studies have reported that cancer-expanded myeloid-derived suppressor cells can directly contact NK cells and induce anergy via membrane-bound TGF-β1 [66], though other studies have found that Treg cells are the main negative regulators of NK cell function in tumours [64]. In our study, anti-TGF-β1 did not completely restore Ca^2+^ flux and cell-cycle arrest, suggesting that some other inhibitory molecules such as IL-10, which is also produced by regulatory CD8^+^ T cells [67], may also play a role in NK impairment during the IT phase of chronic HBV infection. Moreover, recent studies have shown that the partial functional tolerance induced in NK cells by the immunosuppressive cytokine environment in CHB can be corrected *in vitro* by the specific blockage of IL-10 and TGF-β [19]. These findings support our results for IT phase patients and suggest that regulatory CD8^+^ T cells may also influence NK cells in IT-phase patients by producing IL-10. Furthermore, our results demonstrate that the level of IL-10 in IT-phase serum was higher than in sera from healthy controls and IN-phase patients but lower than in sera from IA-phase patients. However, the levels of IL-10 in the sera were significantly lower than the levels of TGF-β1, which suggests that TGF-β1 was the predominant suppressive factor in IT-phase patients.

Our results suggest that the *in vivo* administration of nucleoside analogues partially overcomes the defect in NK cell function in patients. In addition, we observed that NK cell function was significantly lower at the onset of inflammation in IT patients, suggesting that some other inhibitory factors may restrain NK cell function during the initial phase of liver inflammation. Some studies have found that reduced IFN-γ production by NK cells in HBV infection independent of the IT phase [32], we believe this is due to different methods of classifying patient groups. Another interesting finding of our study was the observation that p21 and p15 were obviously elevated on NK cells from IT patients. The cyclin-dependent kinase inhibitors p15 and p21^Waf1/Cip1^ acted as inhibitors of cell-cycle progression [46]. Relatively high levels of Smad-2P and Smad2 were observed in NK cells from IT-phase patients, implying that TGF-β1 had the potential to arrest the NK cell cycle. High rates of p15 methylation in HCCs have been shown to be associated with HCV infection. TGF-β-dependent inhibition of HCV replicons was also associated with cell-cycle arrest in a Smad-dependent manner [68]. Furthermore, the expression of p21 during liver cirrhosis is related to the persistence of infection with HBV [69], which suggests that p21 plays an important role in the progression of HBV. However, one study has shown that the HBV X protein overcomes cellular senescence by down-regulating levels of p16 and p21 via DNA methylation [70]. In that study, HepG2 cells were used, which may explain why their findings differed from our own. Some previous studies reporting persistent HBV infection have focused on genetic variants [71]–[74]. Unfortunately, this study was limited by the supply of freshly isolated NK cells from patients and healthy controls, and therefore, our analysis was restricted to the circulating compartment only. A more detailed investigation of the frequency and function of intrahepatic NK cells in IT patients should be performed.

In summary, our findings demonstrate that high levels of TGF-β1 are associated with reduced NKG2D and 2B4 expression, the functional impairment of NK cell function, and consequently, with the development of persistent HBV. Our study provides a basis for improving current therapies for IT patients by blocking TGF-β1 inhibitory pathways, which could result in additive efficacy at eliminating the virus during the initial phase of CHB infection.

## Materials and Methods

### Ethics statement

Peripheral blood samples and clinical assessments were obtained during routine hepatitis follow-ups. In accordance with the Declaration of Helsinki and the Ethical Board of the Institutional Review Board of the University of Science and Technology of China, all participants provided written informed consent, which was obtained before enrolment in the study.

### Patients, healthy subjects and treatments

Peripheral blood samples were obtained from the Department of Infectious Diseases of the First Affiliated Hospital of Anhui Medical University. The patient characteristics are listed in Table 1. There were no significant differences in demographic variables (gender/age) between the patient and the healthy control groups (Table 1). Blood samples were processed within 4 h of collection. Among the 249 clinical samples, 65 were diagnosed as IT phase, 48 were diagnosed as IA phase, and 41 were diagnosed as IN phase. The diagnostic criteria for the clinical terms of HBV infection were adopted from the National Institutes of Health (NIH) conferences on the management of hepatitis B in 2000 and 2006 [4], [10]. Detailed characteristics of the patients are shown in Table S1. Peripheral blood samples obtained from 95 healthy donors at Hefei Blood Bank and Anhui Provincial Hospital served as controls. All patients were negative for anti-hepatitis C and anti-human immunodeficiency virus antibodies and were treatment naïve. All patients were treated with anti-viral therapy consisting of nucleoside analogues (lamivudine, adefovir dipivoxil, and entecavir) without immunomodulators.

**Table 1:** Clinical characteristics of the enrolled subjects.

Group	HC	IT	IA	IN
Cases	95	65	48	41
Sex (male)	49 (51.6%)	34 (52.3%)	36 (75.0%)	15 (36.6%)
Age (years)	34.1±1.8	31.6±1.4	32.7±1.4	36.9±1.6
ALT (U/L)	18±7	22±1	268±46	21±1
AST (U/L)	18±6	22±1	167±42	21±1
TB (µmol/L)	----	49±11	91±37	32±14
DB (µmol/L)	----	31±9	76±38	20±10
HBV DNA positive(>2000 IU/mL)	0	65 (100%)	48 (100%)	0
HBsAg positive	0	65 (100%)	48 (100%)	41 (100%)
HBeAg positive	0	65 (100%)	37 (77.1%)	0 (0%)
HBeAb positive	0	0 (0%)	11 (22.9%)	41 (100%)
HBcAb positive	0	65 (100%)	48 (100%)	41 (100%)

Alanine transaminase (ALT); Aspartate transaminase (AST); Total bilirubin (TB); Direct bilirubin (DB).

### Flow cytometric analysis

Fresh human peripheral blood mononuclear cells (PBMCs) were isolated from peripheral blood by Ficoll-Isopaque (Solarbio, China) gradient centrifugation. NK cells were freshly purified from PBMCs by negative selection using magnetic microbead separation kits (Miltenyi Biotec), resulting in >90% purity. Human PBMCs and NK cells were prepared and stained with monoclonal antibodies (mAbs). Fc receptors (FcRs) were blocked using normal mouse serum or rat serum. Abs against the following proteins were used for staining human PBMCs: CD3 (SK7), CD56 (B159), CD16 (3G8), CD27 (M-T271), CD226 (DX11), NKp30 (p30-15), NKp44 (p44-8.1), NKp46 (9E2/NKP46), NKG2D (1D11) (BD PharMingen) and 2B4 (eBioC1.7) (eBioscience). For intracellular cytokine assays, PBMCs were fixed in 2% formaldehyde after surface protein staining. After washing, PBMCs were permeabilised with 0.5% saponin, and the cells were then incubated with FITC–anti-IFN-γ (BD PharMingen) or PE–anti-T-bet (eBioscience). For CD107a analysis, PBMCs were cultured with IL-12 (10 ng/ml, R&D systems) and phycoerythrin-conjugated anti-CD107a (BD Bioscience) for 16 hr. Then, the cells were washed, blocked and stained with surface antibodies for ordinary FACS staining. For direct immunofluorescence staining of human whole blood, the appropriate volume of fluorochrome-conjugated monoclonal antibody was added to 100 µL of whole blood, which was then incubated in the dark at room temperature (20° to 25°C) for 30 min. Next, 2 mL of 1× RBC Lysis Buffer (Biolegend) was added, and the solutions were incubated for 10 min in the dark at room temperature. They were then centrifuged at 500× g for 5 min, and the supernatants were removed. After washing once, 0.5 mL of 1% paraformaldehyde was added to the solution, which was mixed thoroughly. The solutions were stored at 2 to 8°C until analysis. Appropriate isotype controls were used in all experiments to estimate background fluorescence. Stained cells were analysed using a FACSCalibur flow cytometer (Becton Dickinson), and the data were analysed using FlowJo analysis software 7.6.1 (Treestar). For the cell-cycle analysis, cells were seeded in 12- or 24-well plates at 60 to 70% confluency. After a 72-h incubation with patient serum, NK cells were harvested, washed in PBS and fixed overnight in 70% ethanol. The next day, the cells were collected, washed in PBS, incubated at 37°C for 10 min with PBS containing 100 mg/mL RNase A and 25 mg/mL propidium iodide (PI) (Sigma-Aldrich) and analysed using flow cytometry (BD Biosciences, San Jose, CA, USA). Data analysis was performed using ModFit LT software for Win32 3.1 (Verity Software House, PO Box 247, Topsham, ME 04086, USA).

### *In vitro* cell culture, stimulation and transfection

NK cell populations were enriched from whole blood by negative selection using an NK Cell Isolation Kit (Miltenyi Biotec). A total of 1×10^6^ human NK cells were cultured in 24-well plates at 37°C in a 5% CO_2_ incubator. The cells were incubated in medium alone or with TGF-β1 (1 ng/ml; R&D systems) and then with IL-15 (10 ng/ml; PeproTech) plus IL-2 (100 U/ml) for 72 h. In another culture model, NK cells were cultured in the presence of 20% healthy control serum, IT patient serum or IA/IN patient serum with or without anti-TGF-β-neutralising Ab (10 µg/ml; R&D systems) for 72 h. For the detection of T-bet expression, PBMCs were cultured in the presence of IFN-γ (10 ng/ml, R&D systems) and IL-12 (5 ng/ml, R&D systems) for 0, 12, or 72 h. For intracellular IFN-γ detection, PBMCs were cultured with RPMI 1640 supplemented with 10% FBS in the presence of IL-12 (10 ng/ml, R&D systems) for 12 h. And then monensin (10 µg/ml, Sigma) was added to prevent the secretion of the induced cytokines for 4 h. Transfection of NK92 cells was performed using an Amaxa Cell Line Nucleofector Kit R VCA-1001(Lonza Swiss). The transfection procedure was performed according to the manufacturer's instructions. Unless otherwise indicated, cells were transfected with 200 nM siRNAs. siRNA duplex homologous in sequence to HCST-119,214,356 and SH2D1A-459,501,674, as well as scrambled negative control constructs, were synthesised and purified by Shanghai Gene-Pharma Co. (Shanghai, China). The primers been used are as follows: HCST-homo-119: (5′- UCC AUG UGG GUC ACA UCC UTT AGG AUG UGA CCC AGA UGG ATT-3′); HCST-homo-214: (5′- GGC ACU UCA GGC UCU UGU UTT AAC AAG AGC CUG AAG UGC CTT-3′); HCST-homo-356: (5′- GGC AAA GUC UAC AUC AAC ATT UGU UGA UGU AGA CUU UGC CTT-3′); SH2D1A-homo-459: (5′- AGG CGU GUA CUG CCU AUG UTT ACA UAG GCA GUA CAC GCC UTT-3′); SH2D1A-homo-501: (5′- CAC GGU UAC AUU UAU ACA UTT AUG UAU AAA UGU AAC CGU GTT-3′) and SH2D1A-homo-674: (5′- GUC CUC AGC UAG AAG UAC ATT UGU ACU UCU AGC UGA GGA CTT-3′).

### Determination of serum cytokine concentrations

Serum cytokine concentrations were analysed using a CBA Inflammation Kit (BD Biosciences) according to the manufacturer's protocols. In brief, 50 µl of patient serum or a standard recombinant protein dilution was added to a mixture of capture beads coated with mAb to a group of cytokines (IL-1α, IL-1β, IL-2, IL-4, IL-6, IL-10, IL-12p70, IL-13, TNF and IFN-γ) and a PE-conjugated detection reagent. After 3 h, the capture beads were washed and acquired by a flow cytometer (BD Biosciences, San Jose, CA, USA). Using the recombinant standards and FlowJo analysis software 7.6.1 (Treestar), cytokine concentrations were quantified for each serum sample. Serum TGF-β1 was assayed using a standard sandwich ELISA Kit (CSB-E04725h) (Cusabio), in which 80 µl of patient serum was analysed according to the manufacturer's high sensitivity protocol. To activate latent TGF-β1 to the immuno-reactive form, serum was incubated with 1N HCL for 10 min at room temperature.

### Analysis of mRNA expression by real-time quantitative RT-PCR

Total RNA was prepared from NK cells from healthy controls and HBV patients using TRIzol Reagent (Invitrogen, CA) according to the manufacturer's instructions. Cellular RNA was used for cDNA synthesis. Semi-quantitative real-time RT-PCR was performed using a SYBR Premix Ex Taq Perfect Real Time Kit (TaKaRa) and a sequence detector (Rotor Gene 3000, Corbett Research). The oligonucleotide primers used to amplify DAP10 were DAP10-F (5′-GGC TGC AGC TCA GAC GAC-3′) and DAP10-R (5′- AGG AGC GGC AGA GAG AGG-3′). The primers used to amplify SAP were SAP-F (5′-GAC GCA GTG GCT GTG TAT-3′) and SAP-R (5′-TCA TGG GGC TTT CAT TTC AGG CAG ACA TCA GG-3′). The primers used to amplify EAT-2 were EAT-2-F (5′-AGA CAG CGA GTC GAT ACC AG-3′) and EAT-2-R (5′-CCG TGT TTC TCT CTG AAG ATT CG-3′). A negative control without cDNA template was performed with every assay. The transcript copy number per subject was calculated by normalisation to GAPDH expression.

### Immunofluorescence analysis

Primary NK cells (5×10^4^ cells) were allowed to adhere to poly-L-lysine-coated slides before fixation in 4% paraformaldehyde for 20 min. Slides were then washed with TBST buffer (10 mM Tris-HCl, 0.1% Tween-20, 150 mM NaCl, pH 7.5) three times for 5 min each and permeabilised with 0.5% saponin (Sigma-Aldrich). After blocking with 1% bovine serum albumin (BSA) in TBS for 1 h, the slides were then washed three times in TBST for 5 min and incubated with a primary antibody (anti-DAP10 (FL-93) sc-25623; anti-SAP (C-18) sc-8639, Santa Cruz Biotechnology) diluted 1∶100 for 1 h. Afterwards, the slides were washed three times again in TBST for 5 min, incubated for 30 min with the secondary antibody (donkey anti-goat IgG-FITC, sc-2024 or goat anti-rabbit IgG-FITC, sc-2012, Santa Cruz Biotechnology) at a dilution of 1∶1,000 and stained with DAPI (Santa Cruz Biotechnology) for 1 min. A final wash in PBS was performed, and the slides were then mounted on coverslips in VECTASHIELD (Vector Laboratories) anti-fade solution. All procedures were performed at room temperature. Images were acquired on a Zeiss 510 Meta multi-photon confocal microscope (Zeiss, Oberkochen, Germany). Analysis was performed using LSM510 META software (Zeiss) and a JD801 Morphological Image Analysis System (JSJD Tech Inc., China). The analysis was performed on five confocal images from 200 cells per experiment in images using channels that showed the co-stained proteins of interest.

### Ca^2+^ flux analysis

NK cells were resuspended (1×10^6^ cells/mL) in culture medium containing 0.2 M Fluo-4 AM (Invitrogen, USA) and 1 M Fura Red AM (Invitrogen, USA) and incubated at 37°C for 30 min. The cells were then washed once with HBSS supplemented with 1% FBS and resuspended at 1×10^6^ cells/mL in 0.5 mL HBSS plus 1% FBS. They were then added to poly-L-lysine-coated (Sigma) wells attached to Lab-Tek II chambered coverslips (Thermo Fisher Scientific Inc.). The NK cells were allowed to settle onto the coverslips at 37°C for 15 min. Then, the medium was replaced with 300 µL cold HBSS plus 1% FBS containing 3 g each of the mAbs specific for NK receptors. The cells were incubated on ice for 30 min. The cells were then washed once in 1 mL HBSS plus 1% FBS, and 300 µL cold HBSS with 1% FBS was placed into each chamber. The chamber was warmed in a 37°C, 5% CO_2_ incubator for 5 min. Data were captured on a Zeiss LSM510 confocal microscope using a 20× objective (Zeiss, Oberkochen, Germany). The microscope was set to scan mode to acquire wavelengths from 499 to 670 nm in a single pass, and the scan speed was set to ensure that one scan was completed every 4 s. Sixty seconds after the beginning of the acquisition process, secondary cross-linking goat anti-mouse F(ab')2 antibody (Jackson Immuno Research, West Grove, PA) was added at a final concentration of 13 µg/mL. Scanning continued for 10 min. The data were analysed by linear unmixing, using control spectra acquired from cells loaded with either Fluo-4 or Fura-Red. After unmixing, the cells were processed to yield ratios of Fluo-4 fluorescence to Fura Red fluorescence for the entire time series.

### Cytotoxicity assay

The cytotoxicity of primary NK cells or NK92 cells against target cells was measured using a standard 4-h ^51^Cr release assay, as previously described [75]. Primary NK cells or NK92 cells were used as effector cells. ^51^Cr-labelled K562 cells (an NK cell–sensitive erythroleukaemia cell line) were used as target cells at effector-to-target cell (E∶T) ratios of 20∶1, 10∶1, and 5∶1. Target cells (1×10^6^) were labelled with 200 µCi sodium [^51^Cr] chromate (PerkinElmer) for 1 h at 37°C and then washed 3–4 times with PBS. Individual assays were performed in triplicate with 1×10^4^ target cells in 96-well V-bottom plates. Maximum ^51^Cr release was determined by incubating target cells with 2% Triton X-100. For spontaneous ^51^Cr release, the targets were incubated without effector cells in assay medium alone. All samples were assayed in triplicate. Plates were briefly spun down and incubated at 37°C for 4 h. After 4 h, the cells were pelleted, and 100 µl of supernatant was counted by a gamma counter. The percentage of specific ^51^Cr release was calculated using the formula ([experimental release-spontaneous release]/[maximum release-spontaneous release])×100%. Spontaneous release did not exceed 10% of the maximum release in all experiments.

### Western blotting

After NK cell purification using MACS (magnetic-activated cell sorting), NK cell lysates were separated by SDS-PAGE under non-reducing conditions on a 12% polyacrylamide gel. After electrophoresis, the proteins were transferred onto PVDF membranes (Millipore) by electroblotting using vertical buffer tanks. The membranes were blocked with blocking buffer (5% non-fat dry milk in wash buffer (50 mM Tris-HCl, pH 7.4, 0.9% NaCl, and 0.1% Tween 20)) overnight at 4°C and then incubated with the primary antibodies (at the recommended dilutions) for 1.5 h at room temperature. The membranes were then washed with wash buffer six times and incubated with HRP-conjugated secondary antibodies for 1 h. After washing six times with wash buffer, protein bands were visualised using an enhanced chemiluminescent system (Pierce). The primary antibodies used [(Smad2 (D43B4), Phospho-Smad2 (Ser465/476), p15^INK4B^ and p21^Waf1/Cip1^ (DCS60)] were all obtained from Cell Signaling Technology.

### Statistical analysis

The data are expressed as the mean ± SEM. One-way ANOVA was used to compare the differences among three or more groups, followed by the Bonferroni *post hoc* test. The analysis was completed using SPSS for Windows (version 10.1; SPSS). In all analyses, P-values<0.05 were considered statistically significant.

### Accession numbers

NKG2D, P26718 (NKG2D_HUMAN); CD244, Q9BZW8 (CD244_HUMAN); DAP10, Q9UBK5 (HCST_HUMAN); SAP, O60880 (SH21A_HUMAN); EAT-2, O14796 (SH21B_HUMAN); Smad2, Q15796 (SMAD2_HUMAN); P15, P42772 (CDN2B_HUMAN); P21, P38936 (CDN1A_HUMAN).

## Supporting Information

Figure S1 **The FACS gating strategy.** (A) The FACS gating strategy for excluding dead and irrelevant cells. (B) The FACS gating strategy for isolating total CD3^+^ CD56^−^ NK cells within the lymphocyte gate.
(TIF)

Figure S2 **The frequency of the expression of other NK cell activation receptors on NK cells from healthy controls and CHB patients.** (A) NKp30, NKp44, NKp46, CD16, CD27 and CD226 expression on total CD3^+^ CD56^−^ NK cells within the lymphocyte gate from a representative healthy control subject (HC) and patients in the immune tolerant phase (IT), immune active (Clearance) phase (IA) and inactive phase (IN). (B) Cumulative data are shown.
(TIF)

Figure S3 **The percentage of NKG2D and 2B4 levels were lower in females than male.** (A) Differential NKG2D and 2B4 expression on total NK cells within the lymphocyte gate in females and males. (B) Differential total CD3^+^ CD56^−^ NK cells within the lymphocyte gate in HC, IT, IA and IN patients.
(TIF)

Figure S4 **T-bet expression in NK cells from patients and healthy controls.** (A) Fresh PBMCs were stimulated with IL-12 and IFN-γ, as descibed in the Materials and Methods. After 0, 12, and 72 h, T-bet expression was determined using flow cytometry by gating on CD3^−^ CD56^+^ NK cells. A representative dot plot of T-bet staining in NK cells is shown. Cumulative data are shown (B).
(TIF)

Figure S5 **Reduced expression of SAP in NK cells from IT patients.** Freshly isolated NK cells obtained from the peripheral blood of healthy controls and HBV patients were analysed by western blotting, and the results for SAP are shown.
(TIF)

Table S1 **Detailed clinical characteristics of patients.** Alanine transaminase (ALT); Aspartate transaminase (AST); Total bilirubin (TB); Direct bilirubin (DB); Serum albumin and globulin ratio (A/G); HBV-5M: HBsAg (1); HBsAb (2); HBeAg (3); HBeAb (4); HBcAb (5).
(XLSX)
