# Supplementary figures and images for: TGF-β1 Down-Regulation of NKG2D/DAP10 and 2B4/SAP Expression on Human NK Cells Contributes to HBV Persistence
Source: PLoS Pathog. 2012 Mar 15;8(3):e1002594. doi: 10.1371/journal.ppat.1002594 (PMC3305436; doi:10.1371/journal.ppat.1002594)

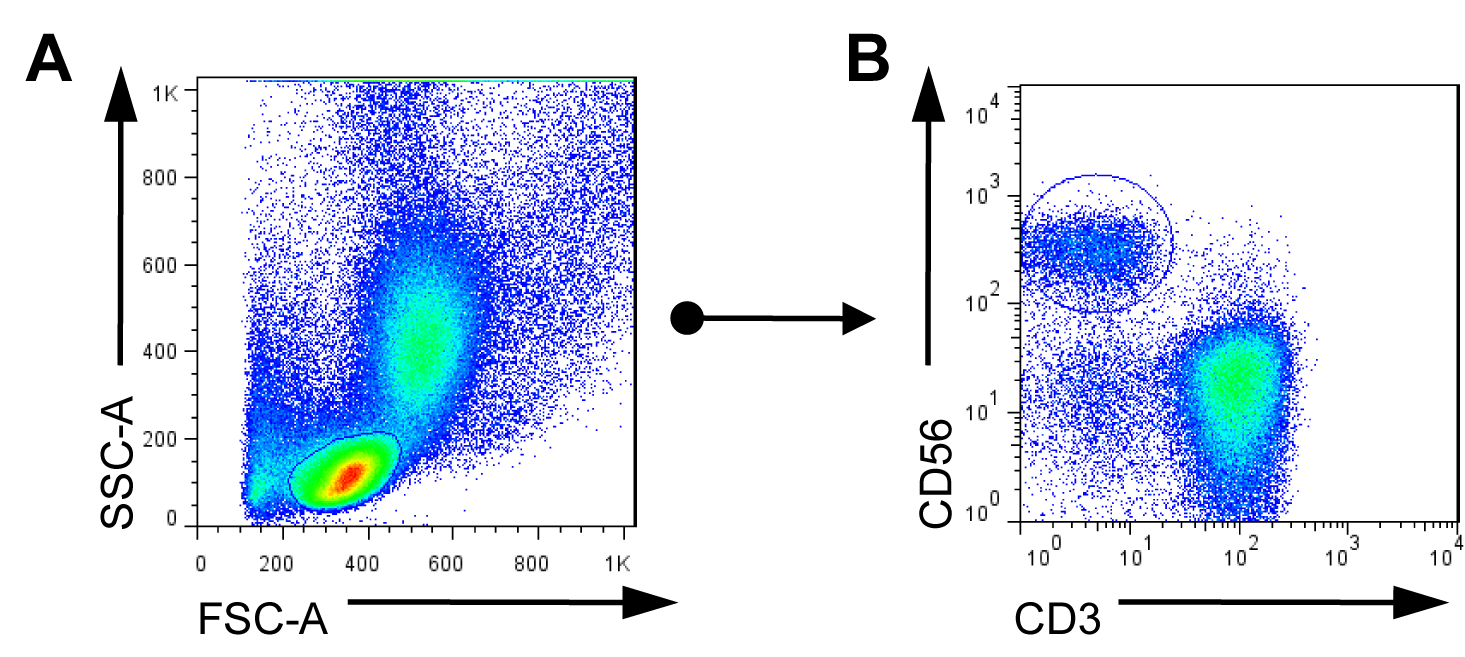

Supplement: Figure S1 — The FACS gating strategy. (A) The FACS gating strategy for excluding dead and irrelevant cells. (B) The FACS gating strategy for isolating total CD3+ CD56− NK cells within the lymphocyte gate. (TIF) [file ppat.1002594.s001.tif]

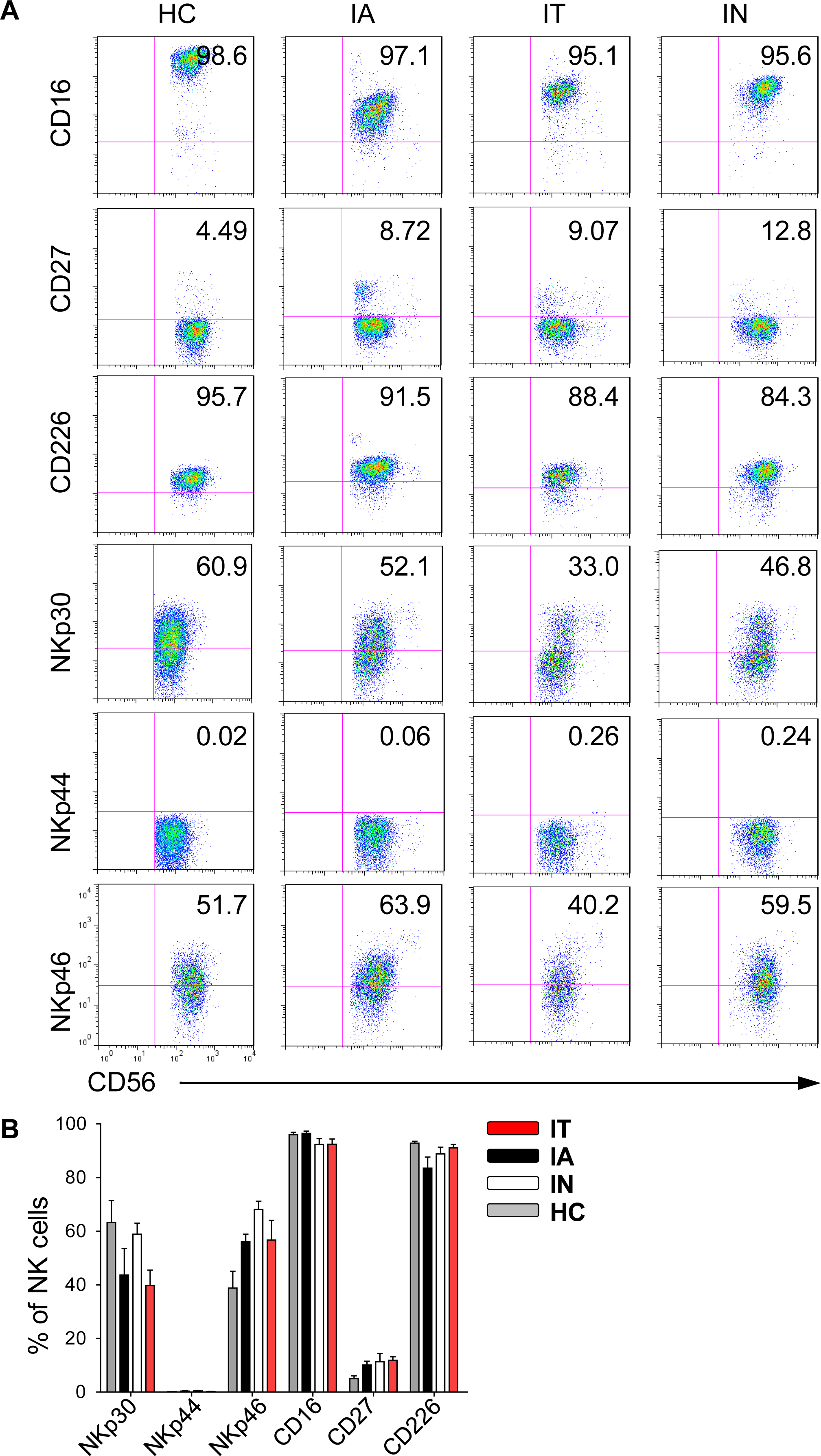

Supplement: Figure S2 — The frequency of the expression of other NK cell activation receptors on NK cells from healthy controls and CHB patients. (A) NKp30, NKp44, NKp46, CD16, CD27 and CD226 expression on total CD3+ CD56− NK cells within the lymphocyte gate from a representative healthy control subject (HC) and patients in the immune tolerant phase (IT), immune active (Clearance) phase (IA) and inactive phase (IN). (B) Cumulative data are shown. (TIF) [file ppat.1002594.s002.tif]

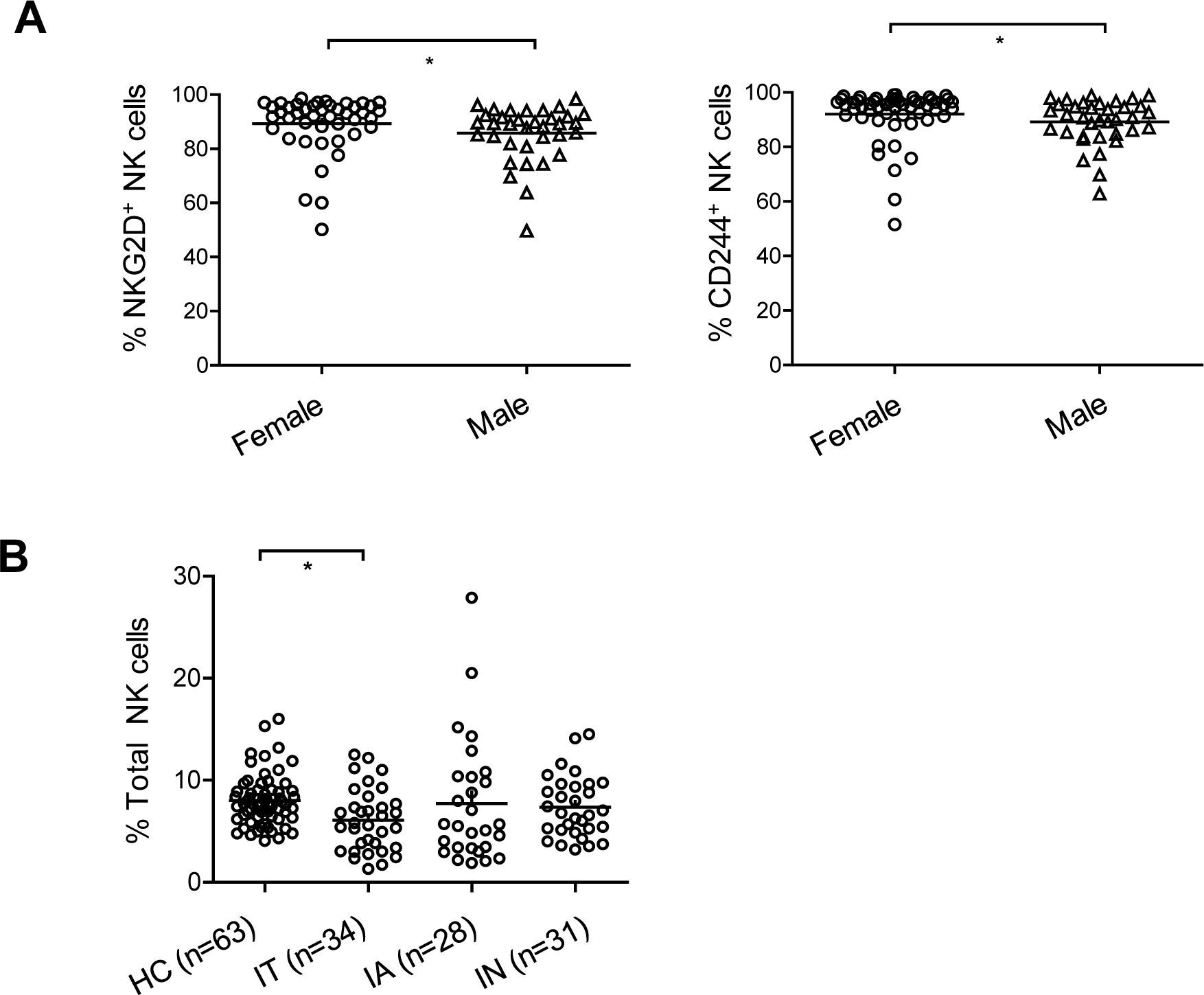

Supplement: Figure S3 — The percentage of NKG2D and 2B4 levels were lower in females than male. (A) Differential NKG2D and 2B4 expression on total NK cells within the lymphocyte gate in females and males. (B) Differential total CD3+ CD56− NK cells within the lymphocyte gate in HC, IT, IA and IN patients. (TIF) [file ppat.1002594.s003.tif]

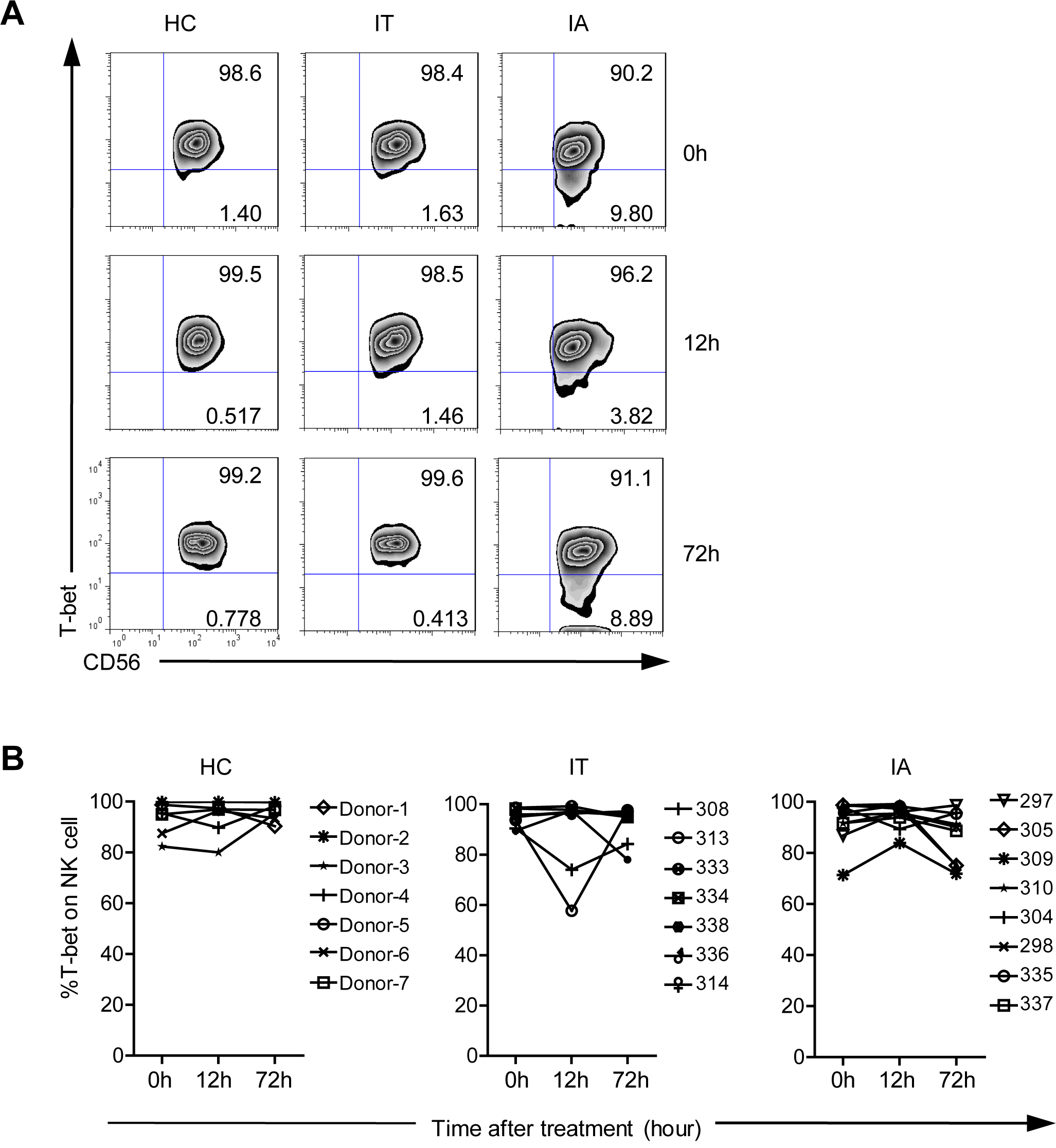

Supplement: Figure S4 — T-bet expression in NK cells from patients and healthy controls. (A) Fresh PBMCs were stimulated with IL-12 and IFN-γ, as descibed in the Materials and Methods. After 0, 12, and 72 h, T-bet expression was determined using flow cytometry by gating on CD3− CD56+ NK cells. A representative dot plot of T-bet staining in NK cells is shown. Cumulative data are shown (B). (TIF) [file ppat.1002594.s004.tif]

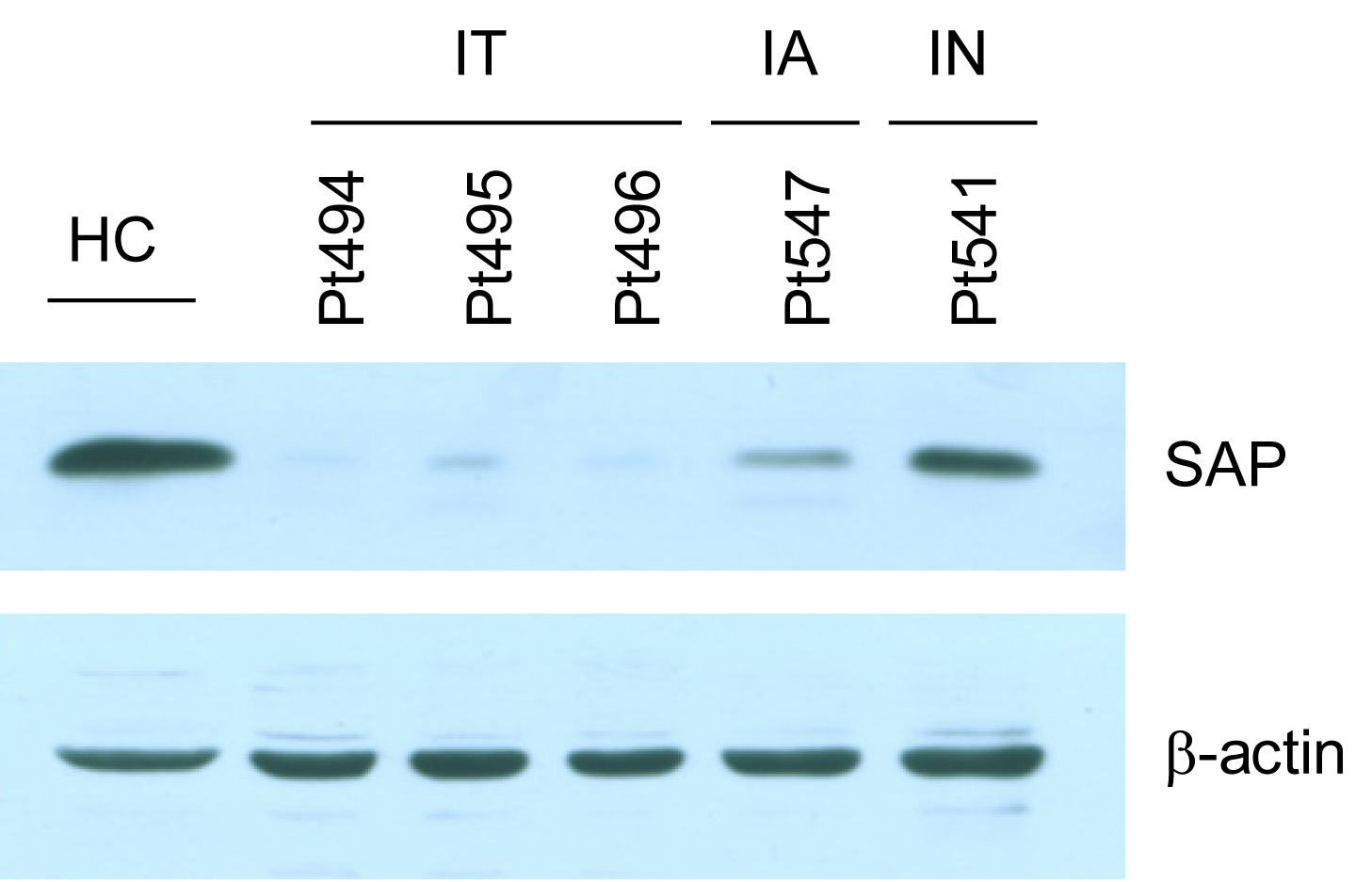

Supplement: Figure S5 — Reduced expression of SAP in NK cells from IT patients. Freshly isolated NK cells obtained from the peripheral blood of healthy controls and HBV patients were analysed by western blotting, and the results for SAP are shown. (TIF) [file ppat.1002594.s005.tif]
